# Retraction: Sanitation, water, energy use, and traffic volume affect environmental quality: Go-for-green developmental policies

**DOI:** 10.1371/journal.pone.0311784

**Published:** 2024-10-14

**Authors:** The PLOS ONE Editors

This article [1] was identified as one of a series of submissions for which PLOS has concerns about adherence to journal policies on authorship and systematic manipulation of the publication process.

In addition, the corresponding author stated that the dataset downloaded from the World Development Indicator Database and used in this study is no longer available.

A member of the Editorial Board reviewed the article and data from the version of the World Development Indicator Database available at the time of post-publication follow-up [2]. They noted concerns including missing observations for many variables across large parts of the study period, lack of discussion of extrapolation methods used, and large discrepancies between data held in the database and the descriptive statistics reported [1]. These issues call into question the validity and reliability of the reported results.

The *PLOS ONE* Editors retract this article because of the above concerns. We regret that the issues were not identified prior to the article’s publication.

KZ and HS did not agree with the retraction and stand by the article’s findings. LK, SA, KH, I, ZBH, and MKA either did not respond directly or could not be reached.
